# Functional Outcome of Distal Femur Nail Multilocking (DFN-ML) in Distal Third Femur Fractures

**DOI:** 10.7759/cureus.92047

**Published:** 2025-09-11

**Authors:** Manish Bisht, Chetan Peshin, Sanad Kumar

**Affiliations:** 1 Orthopaedics, Himalayan Institute of Medical Sciences, Dehradun, IND

**Keywords:** dfn-ml, distal femur, fracture, lysholm knee score, orthopaedic

## Abstract

Background

Distal femur fractures (DFFs) are relatively common injuries, particularly in younger individuals due to high-energy trauma and in older individuals due to lower-force events and poor bone quality. Distal femur nail multilocking (DFN-ML) is a less commonly reported method of fixation with potential biomechanical advantages. This method involves the use of an intramedullary nail that has multiple directional locking screws, which provide better stability and fixation compared to conventional plating and nailing methods.

Objective

The present study aimed to evaluate the functional outcome of patients treated with DFN-ML for distal third femur fractures.

Methodology

A total of 31 patients with distal third femur fractures were included in the study. Preoperatively, patients were evaluated to classify the fracture according to the Arbeitsgemeinschaft für Osteosynthesefragen/Orthopaedic Trauma Association (AO/OTA) classification. Range of movement exercises were started from postoperative day (POD) 3, and partial weight-bearing was started from POD 4. Functional outcomes were assessed using the Lysholm Knee Scoring Scale at four, eight, and 12 weeks postoperatively.

Results

The mean age of the patients was 43.55±21.52 years. The majority of the patient population was male (71%). Road traffic accident (RTA) was the most common injury mode reported in 58.1% of cases. The mean Lysholm score increased significantly from four weeks (22.10±13.41) to eight weeks (41.06±15.28) and 12 weeks (80.65±12.24). Postoperative complications were observed in six (19.4%) patients.

Conclusion

The DFN-ML technique appears to offer sufficient stability, high union rates, and early mobilization for the treatment of DFFs. Our findings suggest that DFN-ML is a viable method that may promote fracture healing by providing robust fixation and enabling early mobilization. Larger multicentric studies with longer follow-up are recommended to validate these results.

## Introduction

Distal femur fractures (DFFs) are relatively common injuries, particularly in younger individuals due to high-energy trauma and in older individuals due to lower-force events and poor bone quality [1]. DFFs make up 6% of all femoral fractures and are expected to occur 10 times per 100,000. These injuries significantly increase morbidity and mortality, particularly in the elderly [2]. They are especially vulnerable to damage to the popliteal vessels due to their close proximity to neurovascular structures [3].

DFFs are classified as supracondylar fractures (SCF), intercondylar fractures, and condylar fractures. SCF are the most common type, occurring just above the knee joint. Intercondylar fractures occur within the intercondylar fossa, whereas condylar fractures involve either the medial or lateral femoral condyle [4]. DFFs have been managed with a variety of methods. Skeletal traction was used to treat patients in bed in the past. The majority of treatment for DFFs in more recent times has been surgical intervention. The following options are utilized: arthroplasty, distal femur replacement, intramedullary nail, distal femur locking plate, external fixator (EX-fix), dynamic condylar screw (DCS), 95-degree angle blade plate (ABP), low-contact dynamic compression plate (LC-DCP), and point contact fixator (PC-FIX) [2].

In conventional plating, stability is achieved by compressing bone fragments together with screws. In contrast, modern locking plates achieve stability through fixed-angle screw-plate constructs, which do not rely on fragment compression but rather provide angular stability and are particularly useful in osteoporotic bone. Nail fixation involves the insertion of an intramedullary nail into the medullary canal to stabilize the fracture, secured by proximal and distal locking screws [5]. Distal femur nail multilocking (DFN-ML) is a relatively newer fixation technique in which multiple directional locking options provide enhanced stability and help distribute load more evenly across the bone, thereby reducing stress concentrations and improving biomechanical performance [6].

DFN-ML fixation has several advantages over conventional plating and nailing methods. One advantage is the ability to achieve stable fixation with minimal tissue dissection, reducing the risk of infection and promoting faster healing. DFN-ML fixation also provides better resistance to torsional forces, which are common in DFFs. Additionally, the multilocking screws of the DFN-ML plate allow for greater control of the bone fragments, reducing the risk of malalignment and promoting faster healing [7]. The present study aimed to evaluate the functional outcomes of patients treated with DFN-ML for distal third femur fractures.

## Materials and methods

Study design

This was a prospective observational study conducted in the Department of Orthopaedics at the Himalayan Institute of Medical Sciences (HIMS), Swami Rama Himalayan University, Dehradun, Uttarakhand, India, during the period from January 1, 2024, to June 30, 2025, following approval from the institute's Ethics Committee (approval number: SRHU/HIMS/ETHICS/2025/68). Adult patients aged 18 years and above, of both sexes, presenting with distal third femur fractures were considered eligible. Fractures classified as Arbeitsgemeinschaft für Osteosynthesefragen/Orthopaedic Trauma Association (AO/OTA) types 32A, 32B, 32C, and 33A were included. Both closed fractures and Gustilo-Anderson type I and II open fractures were managed using DFN-ML after appropriate debridement and antibiotic coverage. Patients younger than 18 years, those with pathological or fragility fractures due to metabolic bone disease, and those with peri-prosthetic fractures were excluded. Polytrauma patients with life-threatening injuries, Gustilo-Anderson type III open fractures, active infections at the fracture site, or associated severe head injuries that prevented early rehabilitation were also not considered for the study.

Study protocol

All patients were evaluated preoperatively, and fractures were classified according to the AO/OTA classification. The same implant, a DFN-ML (Synthes/Johnson & Johnson, New Brunswick, New Jersey, United States), was used in all cases to ensure uniformity. Intravenous antibiotics were administered preoperatively. Patients were positioned supine on a radiolucent table with the knee flexed to 30 degrees. A standard midline incision distal to the inferior pole of the patella was used, and the patellar tendon was retracted laterally. Under fluoroscopic guidance, the guidewire was placed in the intercondylar notch, and an appropriately sized nail was inserted. Distal locking screws were applied in a multilock fashion to enhance stability. Proximal locking was performed as per the manufacturer's guidelines. Postoperatively, intravenous antibiotics were continued for 24 hours, and sutures were removed on postoperative day (POD) 11. Early mobilization was encouraged; range of motion exercises were started on POD 3, and partial weight-bearing was permitted from day 4 onwards.

Follow-up of patients

Patients were followed up at four, eight, and 12 weeks postoperatively. Range of motion exercises were initiated on POD 3 to encourage early mobilization while allowing initial wound healing. Partial weight-bearing (approximately 20-30% of body weight, with the aid of crutches or walker support) was permitted from POD 4 onwards. This standardized rehabilitation protocol was applied to all patients irrespective of fracture type, as stable fixation achieved with DFN-ML allowed for early mobilization. At each follow-up, functional outcomes were assessed using the Lysholm Knee Scoring Scale, and complications such as knee stiffness or infection were recorded.

Lysholm Knee Scoring Scale

The functional results were analyzed utilizing the Lysholm Knee Scoring Scale. Each of the eight items on the scale is assigned a weighted score, and the total is calculated by summing these values. The final score ranges from 0 to 100, with higher scores denoting a better outcome and fewer symptoms or disabilities. Scores are graded as excellent (95-100), good (84-94), fair (65-83), and poor [8].

Statistical analysis

Data were entered using Microsoft Excel (Microsoft Corporation, Redmond, Washington, United States) and analyzed with IBM SPSS Statistics for Windows, Version 27.0 (Released 2019; IBM Corp., Armonk, New York, United States). Categorical variables were expressed as frequencies and percentages, while continuous variables were summarized as mean and standard deviation (SD). The independent t-test was used to compare means between two groups, and the chi-squared test was applied for the comparison of categorical variables. In addition, a one-way analysis of variance (ANOVA) was performed to compare Lysholm scores across different fracture subtypes. A p-value of less than 0.05 was considered statistically significant.

## Results

A total of 31 patients with distal third femur fractures were included in the study. The mean age of the study population was 43.55±21.52 years, which reflects the broad age distribution of patients sustaining DFFs, ranging from young adults to elderly individuals. The majority of the patient population was male (71%), while females made up 29%. Road traffic accident (RTA) was the most common injury mode reported in 58.1% of cases. In the rest of the patients (41.9%), the injury mode was other than RTA (slip and fall, fall of heavy object, etc.). Among the total 31 patients, associated injuries were reported in eight (25.8%) cases. Associated injuries include clavicle fracture (6.5%), distal end radius fracture (6.5%), both bone forearm fracture (3.2%), rib fracture (6.4%), and right humerus fracture (3.2%). Among the 31 patients, 18 (58.1%) had AO type 32A fractures, seven (22.6%) had AO type 32B fractures, two (6.5%) had AO type 32C fractures, and four (12.9%) had AO type 33A fractures (Table 1).

**Table 1:** Sociodemographic details, injury characteristics, and types of fracture RTA: road traffic accident

Variable	Domain	Number
Gender	Male	22 (71%)
	Female	9 (29%)
Mode of injury	RTA	18 (58.1%)
	Other than RTA	13 (41.9%)
Associated injuries	Clavicle fracture	2 (6.5%)
	Distal end radius fracture	2 (6.5%)
	Both bone forearm fracture	1 (3.2%)
	Rib fracture	2 (6.4%)
	Right humerus fracture	1 (3.2%)
Type of fracture	32A	18 (58.1%)
	32B	7 (22.6%)
	32C	2 (6.5%)
	33A	4 (12.9%)

**Figure 1:**
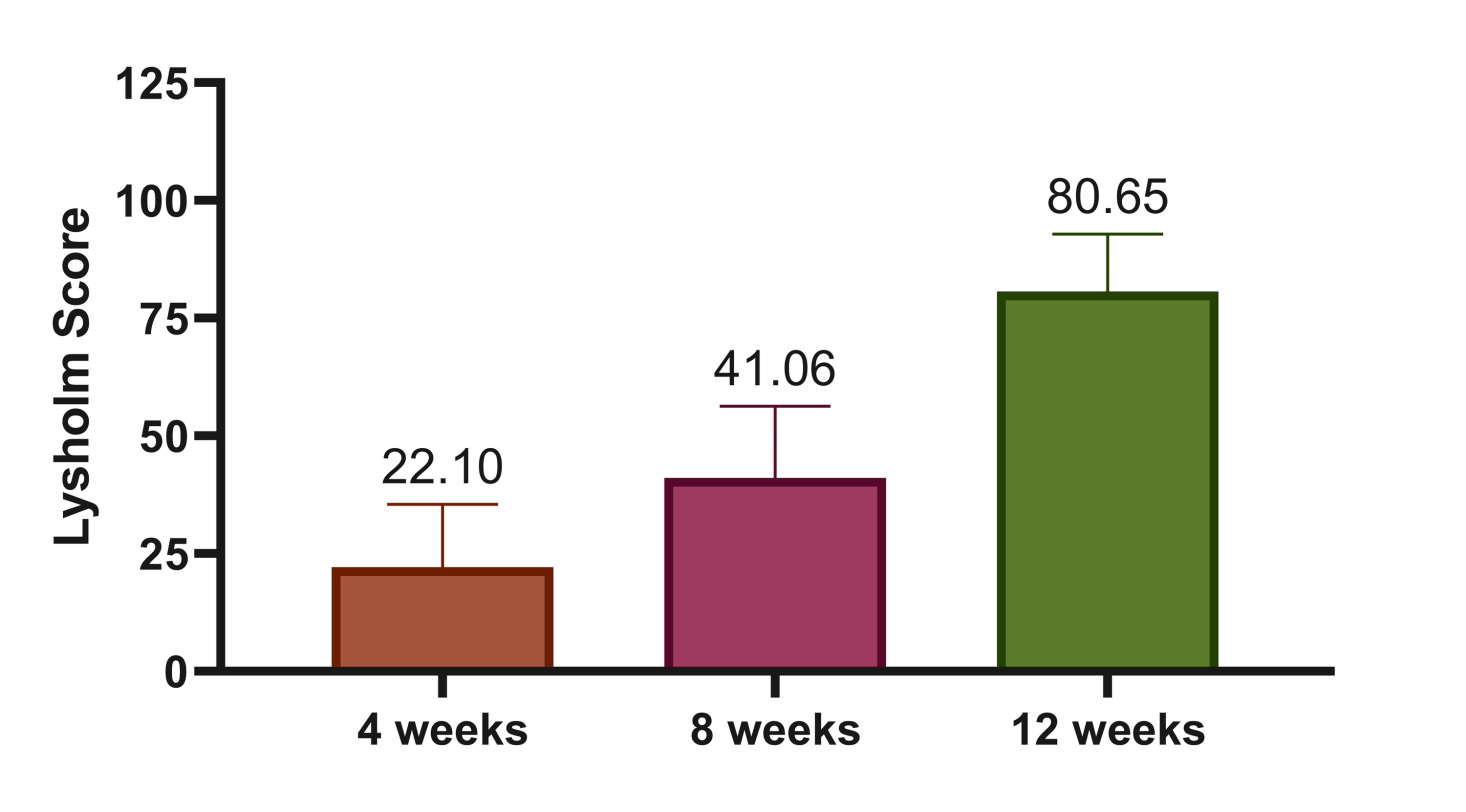
Mean Lysholm score over the follow-up period of 12 weeks

**Figure 2:**
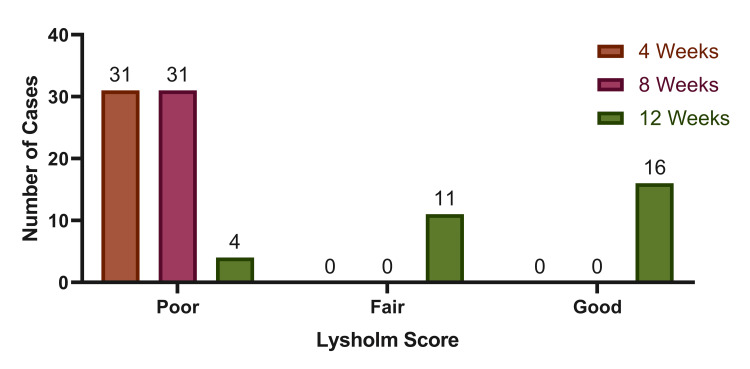
Lysholm score frequency over the follow-up period of 12 weeks

The mean Lysholm score showed a statistically significant improvement over time, increasing from 22.10±13.41 at four weeks to 41.06±15.28 at eight weeks and 80.65±12.24 at 12 weeks (p=0.001) (Figure 1). At four and eight weeks, all 31 patients (100%) had scores in the poor category. By 12 weeks, four patients (12.9%) remained in the poor category, 11 patients (35.5%) had fair outcomes, and 16 patients (51.6%) achieved good outcomes. This distribution demonstrates a progressive and statistically significant shift (p=0.001) from poor to better functional categories over the 12-week follow-up (Figure 2).

Among the total 31 patients, complications were observed in six (19.4%). Knee stiffness occurred in two (6.5%) patients, delayed union was observed in two (6.5%) patients, and infection was noted in two (6.5%) patients (Table 2).

**Table 2:** Complications and types of fracture among patients

Variable	Domain	Number
Complication	Knee stiffness (<90-degree flexion at 12 weeks)	2 (6.5%)
	Delayed union	2 (6.5%)
	Infection (superficial)	2 (6.5%)

**Figure 3:**
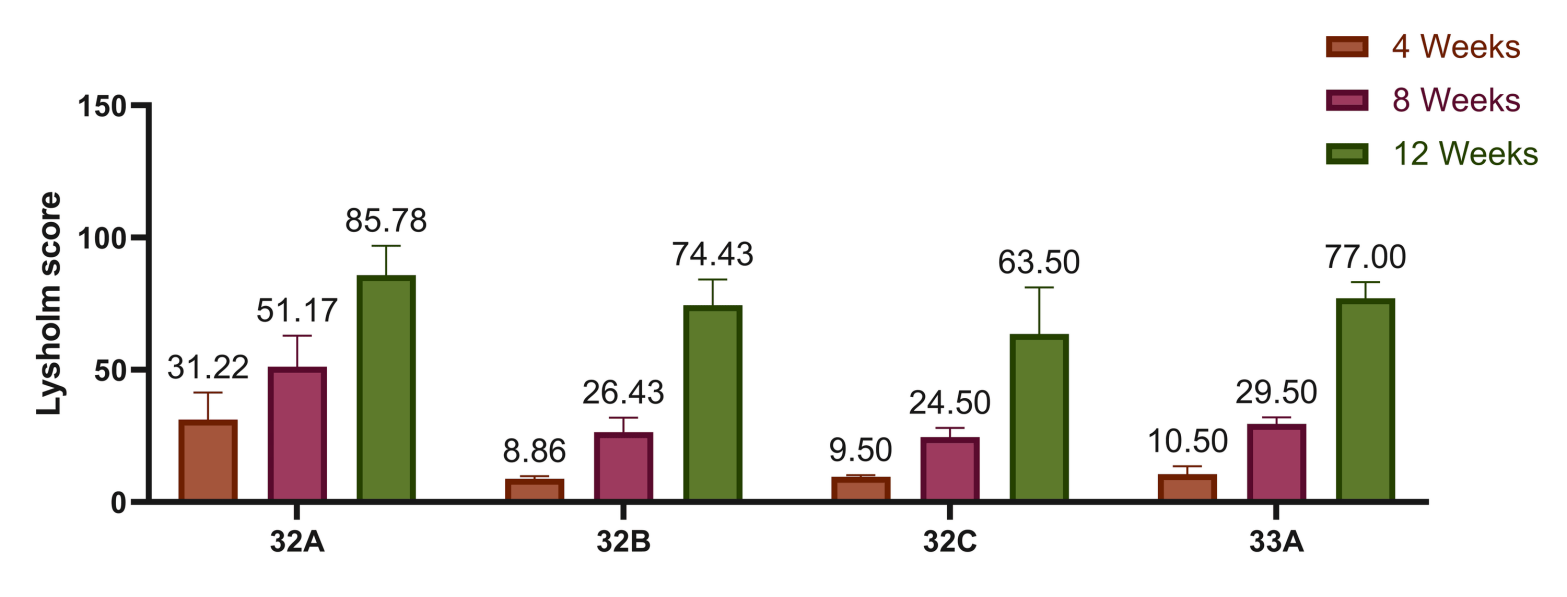
Lysholm score according to the type of fracture

At four weeks, patients with AO type 32A fractures demonstrated a significant improvement in Lysholm scores (p=0.001), followed by those with 33A, 32C, and 32B fractures. A similar pattern was observed at eight weeks, where 32A fractures again showed the most marked improvement (p=0.001). By 12 weeks, improvement remained most pronounced in 32A fractures, followed by 33A, 32B, and 32C. Overall, across the 12-week follow-up, AO type 32A fractures exhibited statistically significant improvement in functional outcome (p=0.018), with a consistent trend of better recovery compared to other subtypes (Figure 3).

**Figure 4:**
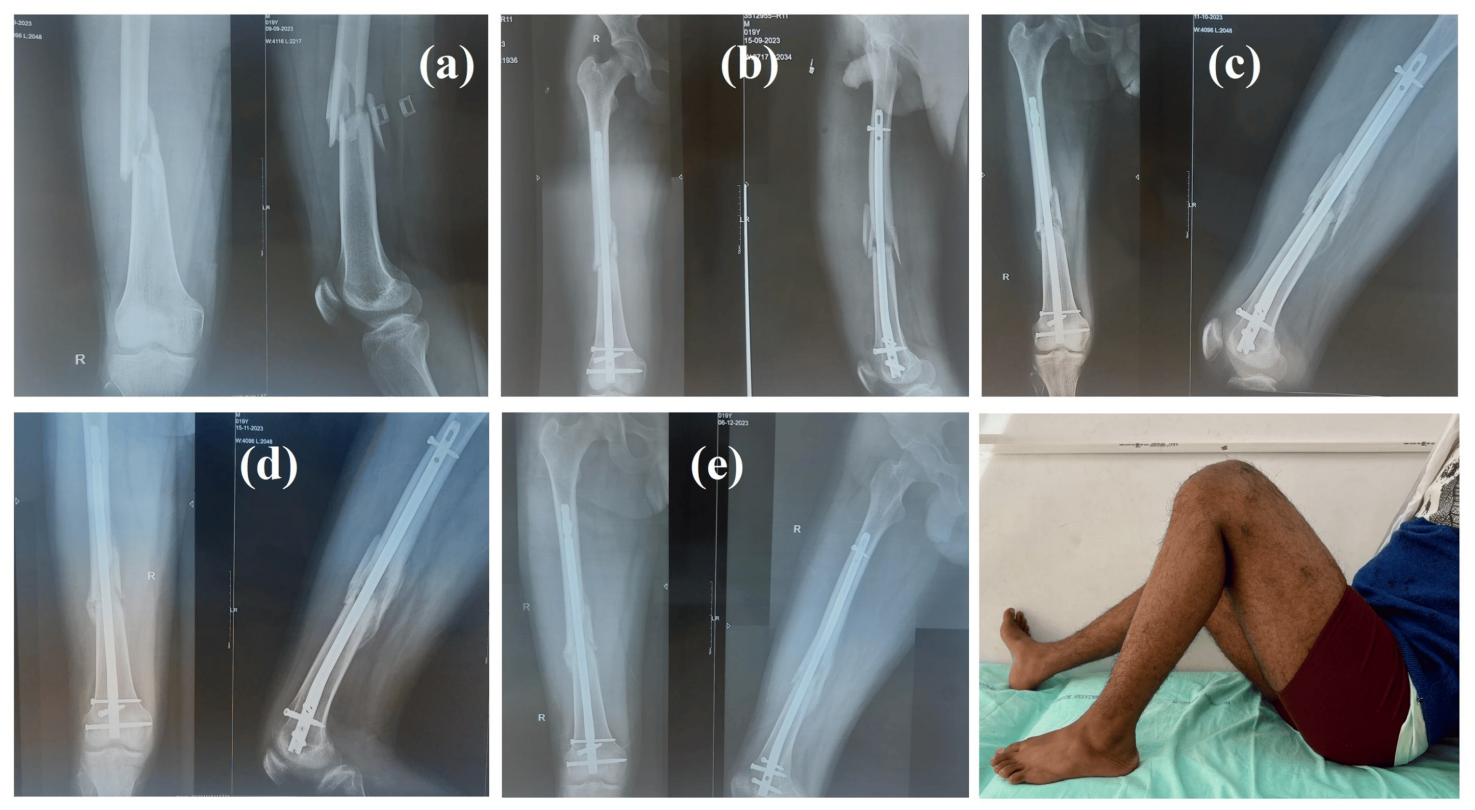
Serial radiographs of a distal third femur fracture treated with DFN-ML (a) Preoperative radiograph showing distal third femur fracture. (b) Immediate postoperative radiograph (POD 1) following DFN-ML fixation. (c) Follow-up at four weeks. (d) Follow-up at eight weeks. (e) Follow-up at 12 weeks demonstrating progressive healing and maintained fixation stability. DFN-ML: distal femur nail multilocking; POD: postoperative day

Serial radiographs confirmed that DFN-ML fixation provided stable alignment and facilitated progressive fracture healing. Early postoperative images showed satisfactory reduction and implant positioning. At four weeks, early callus formation was visible at the fracture margins. By 8-12 weeks, bridging callus was evident with progressive consolidation across the fracture site. The radiographs at the end of follow-up demonstrated continued union with no signs of implant loosening, loss of alignment, or hardware failure. These findings corroborate the functional improvements observed on clinical assessment, highlighting the effectiveness of DFN-ML in maintaining stability and promoting timely union in DFFs (Figure 4).

## Discussion

DFFs are challenging to manage because of their complex anatomy, frequent association with osteoporotic bone, and proximity to vital neurovascular structures. Although locking plates are widely used and provide reliable fixation, intramedullary nails offer comparable advantages such as minimal soft-tissue disruption, effective stabilization in osteoporotic bone, and favorable healing rates. However, there is still no consensus on the optimal method of fixation. The present study was undertaken to evaluate the functional outcomes of DFN-ML as a potential alternative in the management of these fractures.

In the present research, the mean age of subjects was 43.55±21.52 years. Most of the subjects belong to the age group of 21-40 years (45.2%), followed by the age group of >60 years (25.8%) and 41-60 years (19.4%). In the study by Karthik and Kumar, the ages of the patients ranged from 16 years to 83 years with a mean age of 43.8 years [9]. In the study by Kulkarni et al., the age of the subjects ranged from 20 years to 70 years with an average age of 48.7 years [10]. The patients in the Saini et al. research were between the ages of 25 and 72, with a mean age of 45.9 years [11].

In the present research, the majority of the patient population was male (71%), while females made up 29%. In the study by Karthik and Kumar, 4 females and 11 males constituted the study population of 15 patients, indicating a male predominance [9]. In the study by Kulkarni et al., there were 10 women and 20 men [10]. There were 20 (66.66%) male cases and 10 (33.37%) female cases overall in the Saini et al. research [11].

In the present study, RTA was the most common injury mode reported in 58.1% of cases. In the rest of the patients (41.9%), the mode of injury was other than RTA (slip and fall, fall of heavy object, etc.). In the study by Karthik and Kumar, the mechanism of trauma was RTA in 11 patients (73%) and domestic falls in four patients (27%); however, the authors did not specify the fracture classification or age profile for those who sustained fractures after simple falls [9]. According to the study by Elsoe et al., trivial trauma accounted for 61% of DFFs, which the authors attributed to the older age profile and higher life expectancy in the Danish population, leading to a greater incidence of fragility fractures [12]. RTA was the most common cause of injury in the Saini et al. study, affecting 20 patients (66.67%), followed by falls at home (seven patients, 23.33%) and falls from stairs (three patients, 10%) [11].

In the present study, the mean Lysholm score improved significantly from 22.10±13.41 at four weeks to 41.06±15.28 at eight weeks and 80.65±12.24 at 12 weeks (p=0.001). While functional recovery over time is expected in most fixation methods, these results indicate that DFN-ML provided stable fixation that enabled early mobilization and steady improvement in knee function during the follow-up period. At 12 weeks, 51.6% of patients achieved good outcomes and 35.5% had fair outcomes, demonstrating encouraging short-term results. One patient (6.6%) in the Karthik and Kumar trial experienced mild to moderate anterior knee discomfort, which subsided over time with physiotherapy, weight-bearing, and range of motion exercises. Four patients (10.5%) had a 1-2 cm shortening of the operated limb that did not interfere with their everyday activities. Using a modified Hospital for Special Surgery (HSS) knee rating scale, the functional outcome revealed that 46.66% of fractures had outstanding results, 40% had fair results, 6.6% had moderate results, and 6.6% had poor outcomes [9].

In the present study, among a total of 31 patients, complications were observed in six (19.4%) patients. Knee stiffness occurred in two patients (6.5%), delayed union was observed in two patients (6.5%), and superficial infection was noted in two patients (6.5%). Saraglis et al. reported a complication rate of 6.6%. One case of superficial wound infection that occurred two weeks after surgery was successfully treated with a course of oral antibiotics for one week [13]. Two groups were similar in the overall complication rate in the Passias et al. trial [14]. Four patients (26.6%) in the Karthik and Kumar research experienced superficial infections, which were treated for two weeks with intravenous antibiotics [9].

In the present study, among 31 patients, 18 (58.1%) had AO type 32A fractures, seven (22.6%) had 32B, two (6.5%) had 32C, and four (12.9%) had 33A fractures. Similarly, Saini et al. reported that 30% of patients had Müller type A1 fractures, 10% had A3, 26.7% had C1, 23.3% had C2, and 10% had C3 fractures [11]. Papadokostakis et al. also described complication patterns, noting infection in 1.1% of cases, knee septic arthritis in 0.18%, knee discomfort in 16.5%, and malunion in 5.2% [15].

The present study has certain limitations. The first limitation is the relatively small sample size, which is partly due to the low incidence of DFFs. Another limitation is the single-center design, as all patients were recruited from one hospital, which may affect the generalizability of the results. Furthermore, the follow-up period was limited to 12 weeks, allowing only short-term functional outcomes to be assessed. Comorbidities such as diabetes, hypertension, smoking status, and other systemic illnesses were not systematically recorded, despite their known influence on fracture healing and functional recovery, particularly in the Indian population where these conditions are prevalent. Future multicenter studies with larger sample sizes, longer follow-up, and comprehensive comorbidity profiling are warranted to validate and expand upon our findings.

Despite these limitations, the study also has strengths. All patients were treated with the same implant (DFN-ML) and underwent a standardized rehabilitation protocol, which ensured consistency in surgical and postoperative management. Additionally, functional outcomes were prospectively assessed using a validated scoring system (Lysholm Knee Score), providing objective and comparable measures of recovery. These aspects strengthen the reliability of the study's findings and add to the limited literature on DFN-ML in DFFs.

## Conclusions

This prospective study provides preliminary evidence on the short-term functional outcomes of DFFs treated with retrograde DFN-ML. Within 12 weeks of follow-up, DFN-ML allowed stable fixation, early mobilization, and encouraging improvements in knee function. However, given the small sample size, single-center design, and short duration of follow-up, these findings should be interpreted with caution. Larger multicenter studies with longer follow-up and comparative analysis against established fixation methods are needed to validate the role of DFN-ML in the management of DFFs.
